# Numerical aspects of thermo migrated radiative nanofluid flow towards a moving wedge with combined magnetic force and porous medium

**DOI:** 10.1038/s41598-022-14259-x

**Published:** 2022-06-16

**Authors:** Ehsan Ul Haq, Sami Ullah Khan, Tasawar Abbas, Kamel Smida, Qazi Mahmood Ul Hassan, Bilal Ahmad, M. Ijaz Khan, Kamel Guedri, Poom Kumam, Ahmed M. Galal

**Affiliations:** 1 Department of Mathematics, University of Wah, Wah Cantt, 47040 Pakistan; 2 Department of Mathematics, COMSATS University Islamabad, Sahiwal, 57000 Pakistan; 3 Department of General Sciences, College of Applied Sciences, AlMaarefa University, DiriyahRiyadh, 13713 Saudi Arabia; 4 Department of Mathematics and Statistics, Riphah International University, I-14, Islamabad, 44000 Pakistan; 5 Department of Mechanics and Engineering Science, Peking University, Beijing, China; 6 Mechanical Engineering Department, College of Engineering and Islamic Architecture, Umm Al-Qura University, P.O. Box 5555, Makkah, 21955 Saudi Arabia; 7 Center of Excellence in Theoretical and Computational Science (TaCS-CoE) and KMUTT Fixed Point Research Laboratory, Room SCL 802 Fixed Point Laboratory, Science Laboratory Building, Department of Mathematics, Faculty of Science, King Mongkut’s University of Technology Thonburi (KMUTT), 126 Pracha-Uthit Road, Bang Mod, Thung Khru, Bangkok, 10140 Thailand; 8 Department of Medical Research, China Medical University Hospital, China Medical University, Taichung, 40402 Taiwan; 9 Mechanical Engineering Department, College of Engineering, Prince Sattam Bin Abdulaziz University, Wadi addawaser, 11991 Saudi Arabia; 10 Production Engineering and Mechanical Design Department, Faculty of Engineering, Mansoura University, Mansoura, P.O 35516 Egypt

**Keywords:** Engineering, Nanoscience and technology

## Abstract

The researchers are continuously working on nanomaterials and exploring many multidisciplinary applications in thermal engineering, biomedical and industrial systems. In current problem, the analytical simulations for performed for thermos-migration flow of nanofluid subject to the thermal radiation and porous media. The moving wedge endorsed the flow pattern. The heat source effects are also utilized to improves the heat transfer rate. The applications of thermophoresis phenomenon are addressed. The formulated set of expressions are analytically treated with implementation of variational iteration method (VIM). The simulations are verified by making the comparison the numerical date with existing literature. The VIM analytical can effectively tackle the nonlinear coupled flow system effectively. The physical impact for flow regime due to different parameters is highlighted. Moreover, the numerical outcomes are listed for Nusselt number.

## Introduction

Nowadays researchers and engineers are showing great interest to examine nanofluids heat transportation problems. In fact, different working liquids have poor thermal proficiency, and are not favored for heat transportation applications. This problem is overcome by usage of tiny-sized nanoparticles additives. Suspensions of base fluids with small size materials like oxides, metals and carbon nanotubes is considered effective solution for enhancing heat transportation processes. It is experimentally proven that these nanofluids conveys amazing thermal characteristics of base solutions. Nanofluids have emended thermal diffusivity, conductivity, convective heat coefficients and viscosity in comparison with simple liquids like water or oil. Nanoparticles referred important applications in hybrid engines, biosciences, fuels, medical, pharmaceutical, engineering, nanotechnologies and numerous mechanical and chemical industries. The prestigious applications involve power generation, cancer chemotherapy, cooling of devices and reactors, thermal insulation, artificial heart surgery, solar energy absorption, efficiency of chillers and refrigerators etc. Choi^1^ exploited the fundamental thermal aspect for nanomaterials. Turkyilmazoglu^2^ proposed the thermal dynamic of nanofluids by performing the external framework of hydrodynamic and ensured the stability for nanofluids. Nadeem et al.^3^ tested the heat transfer enhancement for the nanofluid referred to the implementation of anisotropic slip effects. Hosseinzadeh et al.^4^ justified the role of microorganisms for three-dimensional nanoparticles flow subject to cross base material. Ramanahalli et al.^5^ observed the continuation of heat transfer for nanofluid flow with Marangoni transport and activation energy. Xiong et al.^6^ computed the thermal observations for the Darcy-Forchheimer flow due to vertical needle carrying the nanoparticles. The optimized slip flow consideration for different nanoparticles was elaborated in the analysis of Xiong et al.^7^. Benos et al.^8^ observed thermal trend for natural convection flow with carbon nanotubes. Gkountas et al.^9^ studied aluminum oxide nanoparticles flow for heat exchangers. Madhukesh et al.^10^ investigated the nanofluid analysis carrying the AA7072 and AA7075 nanoparticles confided by moving curved space. Hamid et al.^11^ discussed the applications of Hall current for ethylene glycol and hybrid nanofluid suspension. Shi et al.^12^ observed the on set of bioconvection for cross nanofluid reflecting the features of activation energy. The biofuel applications based on the microorganism’s flow of couple stress nanofluid was intended by Khan et al.^13^. The rotating cone flow of nanomaterials with entropy generation features was directed by Li et al.^14^.

The mass movement and convective heat of liquids are guaranteed in various thermal engineering applications for instant thermal isolation, refinement of crude oil, heat exchangers and toxic waste disposal^15^. Similarly, boundary layer theory is being used in a variety of engineering fields. The most commonly used of this principle is to define the skin friction drag acting over a body stirring across a stream thus the drag of an aero-plane wing, a blade of turbine or a whole-ship^16^. First time, Falkner and Skan^7^ developed a steady laminar fluid flow model across a static wedge to highlight the applicability of boundary layer Prandtl's principle. In their work, to reduce boundary layer restricted differential equations, Falkner–Skan used similarity transformations to a 3^rd^ order normal nonlinear differential equation. Subsequently, Hartree^8^ used similarity transformation to study the similar problem and provided findings for wall shear stress for various wedge angles, numerically. Later, the hydromagnetic convection at a wedge and a cone was explored numerically by Vajravelu and Nayfeh^9^ In their studies, many intriguing behaviours are shown by the numerical findings for transfer of flow and heat characteristics. Afterward, MHD convection free flow across a field of magnetic with transverse effect along a wedge was examined by Watanabe and Pop^10^. Moreover, Yih^11^ studied non-isothermal wedges and Chamka et al.^12^ inspected the existence of the thermal radiation effect in a non-isothermal wedge along an mean of heat influence. In the happening of heat generation/absorption, Ahmad and Khan^13^ investigated the viscous dissipation impact over a wedge in motion along convection. This work has been studied numerically for numerous values of dimensionless parameters. Various flow regions with different flow fields were examined by Goud and et al.^14,15^. Investigation for the sway of thermal radiation over a MHD stagnation point stream on a slip boundary conditions stretching sheet managed by B.S. Goud^16^. Recently, the mass transfer, joule heating, and effects of Hall current on MHD peristaltic hemodynamics were investigated, through an inclined tapered vertical conduit,^17^. The flow of Casson fluid along transformation of heat with influence upon symmetrical wedge was observed by Mukhopadhyay et al.^18^. The impact of radiation and Hall by heterogeneous convection of Casson fluid flow across a stretched sheet was inspected by Naik et al.^19^. Presently, Bushra et al.^20^ examined the 3D bio convection Casson nanofluid flow flanked by both stretchable and rotating disks. In the occurrence of a magnetic field, Ali and Alim^21^ examined the border-layer study flow of nanofluid via a motile permeable wedge using a falkner skan model. Amar et al.^22^ analysed MHD laminar boundary layer flow through a wedge along the MHD heat and mass transfer impact. Jafar et al.^23^ inquired the wedge movement in a parallel stream along an induced magnetic field. Influence of chemical reactions looked by Kasmani et al.^24^ at convective transmission of heat of a nano fluid boundary layer across a wedge along absorption and suction of heat. Over a permeable stretched wedge, Su et al.^25^investigated impact of Ohmic heating and the thermal radiation. The mixed unsteady boundary layer flow of convection across a chemical reaction on wedge, injection/suction behavior in the context for absorption of heat was explored by Ganapathirao et al.^26^. The flow patterns of the MHD Catto–Christo through wedge and cone subjected to different sources of heat and sinks were scrutinized by Suleman et al.^27^. Rahman et al.^28^ evaluated nanofluid over water passing through a wedge along generation/absorption surface of a convective heat. Khan et al.^29^ studied numerically the effect of thermal radiation on MHD bio-convection flow via a porous wedge. Abbas et al.^30^ investigated the influence of thermal radiation and partial slip through the porous medium on the performance of electrically conducting viscous fluids. A large array of engineers and scientists have investigated the boundary layer nanofluid flow across stretched surfaces under a variety of thermo-physical norms. These problems tend to be more difficult to solve either numerically or analytically and various techniques are implemented^31–37^. The current communication is an improvised account of numerous flow effect of MHD boundary layer through wedge across a porous medium over transfer of heat mass along influence of heat and radiation source.

Following the motivated applications of nanofluids in various engineering and industrial phenomenon, such research endorses the applications of heat source and thermal radiation for the magnetized flow of nanofluid due to moving wedge. The analytical simulations are worked out by using the variational iteration method (VIM). This investigation leads following fulfil the following objectives:

- Present a mathematical model for the wedge flow of nanofluid moving with different angle of rotation.
- The fluid flow is subject to the porous medium and magnetic force impact.
- The heat source and external heating source are implemented for analyzing the heat transfer phenomenon.
- The applications of magneto-nanoparticles are focused with thermos-migration phenomenon.
- A novel variational iteration method (VIM) is followed to simulate the computations^38–41^.
- The accuracy of VIM scheme is checked with against different schemes and verified.
- Some recent advances on fluid flow analysis is listed in Refs.^45–50^.

## Mathematical formulation

Flow of a two-dimensional, laminar boundary layer past a wedge is considered. This flow is incompressible, electrically conducting nanofluid embedded in a porous medium, and the heat transfer effects are caused by the viscous effects. The x-axis is parallel to the plate, while y-axis is in opposition to the free stream. The schematization is based on the coordinate scheme and somatic development described by Amar and Kishan^22^. Taking temperature ($T_{w}$) and nano particles concentration $C_{w}$ are uniform and constant at the wedge wall, correspondingly, are greater than ambient nanoparticles $(C_{\infty } )$ and the ambient temperature $(T_{\infty } )$, in like manner. The physical characteristic of fluid is continuous with fixed magnetic $B_{0}$, normal to wedge wall and used in positive *y*-axis. Since it is so relatively tiny for compared the applicable magnetic field, the induced magnetic field is reason of an electrically conducting fluid in motion is ignored. Using aforementioned assumptions, the governing equations of flux are as follows

1 $$\frac{\partial u}{{\partial x}} + \frac{\partial v}{{\partial y}} = 0$$

2 $$u\frac{\partial u}{{\partial x}} + v\frac{\partial v}{{\partial y}} = u_{e} \frac{du}{{dx}} + v\frac{{\partial^{2} v}}{{\partial y^{2} }} - \left( {\frac{{\sigma B_{0}^{2} }}{\rho }} \right)u,$$

3 $$\begin{gathered} u\frac{\partial T}{{\partial x}} + v\frac{\partial T}{{\partial y}} = \alpha_{f} \frac{{\partial^{2} T}}{{\partial y^{2} }} + \tau \left( {D_{B} \left( {\frac{\partial T}{{\partial y}}\frac{\partial c}{{\partial y}}} \right) + \frac{{D_{T} }}{{T_{\infty } }}\left( {\frac{\partial T}{{\partial y}}} \right)^{2} } \right) + \frac{{Q^{/} }}{{\rho C_{\rho } }}\left( {T - T_{\infty } } \right) + \frac{{V_{f} }}{{C_{\rho } }}\left( {\frac{\partial u}{{\partial y}}} \right)^{2} \hfill \\ + \frac{{16\sigma_{1} T_{\infty }^{3} }}{{3\rho C_{\rho } K_{1} }}\left( {\frac{{\partial^{2} T}}{{\partial y^{2} }}} \right), \hfill \\ \end{gathered}$$

4 $$u\frac{\partial C}{{\partial x}} + v\frac{\partial C}{{\partial y}} = D_{B} \frac{{\partial^{2} C}}{{\partial y^{2} }} + \frac{{D_{T} }}{{T_{\infty } }}\frac{{\partial^{2} T}}{{\partial y^{2} }}$$

The boundary conditions are given as

5 $$\left. \begin{gathered} u - u_{w} (x) = - \lambda u_{e} (x), \, v = 0, \, T = T_{w} , \, C = C_{w} {\text{ for }}y = 0, \hfill \\ u = u_{e} (x), \, T \to T_{\infty } , \, C \to C_{\infty } {\text{ for }}y \to \infty , \hfill \\ \end{gathered} \right\}$$

Here fix moving parameter denoted as $\lambda$, for stretching wedge $\lambda$ is negative, in contrast, $\lambda$ is positive for contracting wedge however,$\lambda = 0$ for static wedge. Here the velocity components is depicted by $(u,v)$ along the $(x,y)$ paths. Similarly $U(x) = U_{\infty } x^{m}$ represent the velocity of fluid at the wedge beyond the boundary layer. Then Eq. (3) can be in the following form:

6 $$\frac{1}{{\rho_{f} }}\frac{\partial p}{{\partial x}} = U\frac{\partial U}{{\partial x}} + \left( {\frac{{\sigma B_{0}^{2} }}{{\rho_{f} }} + \frac{{V_{f} }}{K}} \right)U,$$

Using the Eq. (6) in Eq. (2), we have

7 $$u\frac{\partial C}{{\partial x}} + v\frac{\partial C}{{\partial y}} = U\frac{\partial U}{{\partial x}} + V_{f} \frac{{\partial^{2} u}}{{\partial y^{2} }} + \left( {\frac{{\sigma B_{0}^{2} }}{{\rho_{f} }} + \frac{{V_{f} }}{K}} \right)\left( {U - u} \right),$$

Falkner–Skan power-law parameter is denoted by m, which is aligned with wedge angle, and gradient of Hartree pressure factor $$\, \beta = {\raise0.7ex\hbox{${2m}$} \!\mathord{\left/ {\vphantom {{2m} {(1 + m)}}}\right.\kern-\nulldelimiterspace} \!\lower0.7ex\hbox{${(1 + m)}$}} \,$$ is the demonstrating to $$\, \beta = \frac{\Omega }{\pi }$$ for complete wedge angle^32^. Physically, $$\, m = 1 \,$$ indicates stagnation point, for Blasius solution, positive $$\, m$$ shows pressure gradient, while negative $$m$$ represent adverse pressure gradient.

Now introduce $\psi {\text{(x,y) }}$ as a stream function in such way $u = \frac{\partial \psi }{{\partial {\text{y}}}}{,}v \, = - \frac{\partial \psi }{{\partial {\text{x}}}}$ and the use appropriate the similarity transformation as given:

$$\begin{gathered} \eta = y(\frac{{(1 + m)U_{\infty } }}{{2V_{f} }})^{\frac{1}{2}} x^{{\frac{(m - 1)}{2}}} ,\psi (x,\eta ) = (\frac{{2V_{f} U_{\infty } }}{1 + m})^{\frac{1}{2}} x^{{\frac{(m - 1)}{2}}} f(\eta ),f^{{\prime}} (\eta ) = \frac{u}{v}, \hfill \\ g(\eta ) = \frac{{(T - T_{\infty } )}}{{(T_{w} - T_{\infty } )}},\varphi (\eta ) = \frac{{(C - C_{\infty } )}}{{(C_{w} - C_{\infty } )}}. \hfill \\ \end{gathered}$$

Using the above transformations, in Eqs. (2)–(4) whereas Eq. (1) is satisfied identically, then obtained the following ordinary differential equations system as given

8 $$f^{{\prime\prime\prime}} + f^{{\prime}} f^{{\prime\prime}} - \beta (1 - f^{{\prime}2} ) + \frac{M + K}{{1 + m}}[1 - f^{{\prime}} ] = 0,$$

9 $$\frac{(1 + R)}{{\Pr }}g^{\prime\prime} + [fg^{\prime} + Ecf^{^{\prime\prime}2} + Nbg^{\prime}\phi^{\prime} + Ntg^{^{\prime}2} + Qg] = 0,$$

10 $$\varphi ^{\prime\prime} + Le(f\varphi ^{\prime}) + \frac{Nt}{{Nb}}g^{\prime\prime} = 0,$$

The changed boundary conditions are

11 $$\left. \begin{gathered} f(0) = 0,\,\,\,\,\,f^{\prime}(0) = - \lambda ,\,\,\,\,\,g(0) = 1,\,\,\,\,\,\varphi (0) = 1,\, \hfill \\ f^{\prime} \to 1,\,\,\,\,\,\,\,g \to 0,\,\,\,\,\,\varphi \to 1,\,\,\,\,\,\,\,\,\,\,\,as\eta \to \infty \, \hfill \\ \end{gathered} \right\}$$

whereas the values of

$$\begin{gathered} \, M = \frac{{2\sigma B_{0}^{2} x^{1 - m} }}{{\rho_{f} U_{\infty } }}, \, \beta = \frac{2m}{{m + 1}},{\text{ K}} = \, \frac{{2V_{f} x^{1 - m} }}{{\rho_{f} U_{\infty } }}, \, R = \frac{{16\sigma_{1} T_{\infty }^{3} }}{{3\rho C_{\rho } K_{1} v}}, \hfill \\ Ec = \frac{{U^{2} }}{{c_{p} (T_{w} - T_{0} )}}, \, Q = \frac{2Q^{\prime}x}{{3\rho C_{\rho } (m + 1)u_{e} }}, \, Le = \frac{{\alpha_{f} }}{{D_{B} }},\,\Pr = \frac{{V_{f} }}{{\alpha_{f} }}, \hfill \\ Nb = \frac{{\tau D_{B} (C_{w} - C_{\infty } )}}{{V_{f} }},\,\,\,Nt = \frac{{\tau D_{\tau } (T_{w} - T_{\infty } )}}{{V_{f} T_{\infty } }},\,\,{\text{Re}}_{x} = \frac{{Uw_{x} }}{{V_{f} }}, \hfill \\ C_{f} = \frac{{2\tau_{w} }}{{\rho U^{2} (x)}},\,\,Nu_{x} = \frac{{xq_{w} }}{{K(T_{w} - T_{\infty } )}},\,\,Sh_{x} = \,\frac{{xM_{w} }}{{D_{B} (C_{w} - C_{\infty } )}} \hfill \\ \end{gathered}$$

In this work, the physical magnitude of engineering importance are the Nusselt number, local Skin friction coefficient and local Sherwood number, and are designed as the surface heat flux, mass flux and shear stress, correspondingly, they are given as:

12 $$\tau_{W} = \mu_{F} \left( {\frac{\partial u}{{\partial Y}}} \right)_{Y = 0} \, M_{w} = - D_{B} \left( {\frac{\partial C}{{\partial y}}} \right)_{y = 0} , \, q_{w} = - K_{f} \left( {\frac{\partial T}{{\partial y}}} \right)_{y = 0} .$$

The dimensionless rates of velocity, temperature and concentration are categorized as

13 $$C_{fx} {\text{Re}}_{x}^{1/2} = \, - 2\sqrt {(\frac{m + 1}{2})} f^{\prime\prime}(0), \, \,\,\frac{{Nu_{x} }}{{{\text{Re}}_{x}^{1/2} }} = - \sqrt {(\frac{m + 1}{2})} g^{\prime}(0),\,\,\frac{{Sh_{x} }}{{{\text{Re}}_{x}^{1/2} }} = - \sqrt {(\frac{m + 1}{2})} \varphi ^{\prime}(0),$$

## Variational iteration method (VIM)

For the first time He in 1999^42^ established the Variational Iteration Method is the comprehensive, simple and user friendly technique to solve the differential equations. It has been extensively applied by many researchers to solving problems with high non-linearity. He used this technique for approximate finding for non-linear differential equations.The general form differential equation is pondered as:

14 $$Lv + Nv = g(x),$$

In above equation $v$ is unknown function which is to be determined, linear operator is $L$ and nonlinear linear is $N$, similarly the inhomogeneous term is $g(x)$. The correction functional for above equation^8–11^ can form as

15 $$v_{n + 1} = v_{n} (x) + \int\limits_{0}^{x} {\lambda (t)\left[ {Lv_{n} (t) + Nv_{n} (t) - g(t)} \right]dt,}$$

where in above equation the Lagrange's multiplier is λ is and it may be a constant or a functions. In this method, first we determine the value of Lagrange multiplier which may be determined optimally by using restricted variation and through integration by parts. By using the value of Lagrange multiplier^13^ determine the $v_{n + 1} (x)$ as successive approximations of the solution $v(x).$ The zero-th ordered approximation $v_{0} (x)$ can be any selective function. Finally the solution is

16 $$v(x) = \mathop {\lim }\limits_{n \to \infty } v_{n} (x).$$

### Solution Procedure with VIM

We express $f(\eta ),g(\eta ){\text{ and }}\varphi (\eta )$ functions into the following form of base functions

17 $$\{ \eta^{n} ;n \ge 0\} ,$$

In the form

18 $$f(\eta ) = \sum\limits_{n = 1}^{\infty } {a_{n} \eta^{n} } ,g(\eta ) = \sum\limits_{n = 1}^{\infty } {b_{n} \eta^{n} } ,\varphi (\eta ) = c_{0} + \sum\limits_{n = 1}^{\infty } {c_{n} \eta^{n} } ,$$

Here $a_{n} ,b_{n} {\text{ and }}c_{n}$ are the coefficients to be decided. To apply Variational iteration method we select the initial guesses as follows:

19 $$f_{0} (\eta ) = - \lambda \eta + \frac{1}{2}A_{1} \eta^{2} ,$$

20 $$g_{0} (\eta ) = 1 + A_{2} \eta$$

21 $$\varphi_{0} (\eta ) = 1 + A_{3} \eta ,$$

According to VIM the correction functions are given by as follows:

22 $$f_{n + 1} (\eta ) = f_{n} (\eta ) + \int_{0}^{\eta } {\lambda_{f} (s)\left[ {f_{n}^{{\prime\prime\prime}} + f_{n}^{^{\prime}} f_{n}^{{\prime\prime}} - \beta (1 - f_{n}^{{\prime}2} ) + \frac{M + K}{{1 + m}}[1 - f_{n}^{{\prime}} ]} \right]ds,}$$

23 $$g_{n + 1} \left( \eta \right)\, = \,\,g_{n} (\eta ) + \int_{0}^{\eta } {\lambda_{g} (s)\bigg[\frac{(1 + R)}{{\Pr }}g_{n} ^{\prime\prime} + [f_{n} g_{n} ^{\prime} + Ecf_{n} ^{{\prime\prime}{2}} + Nbg_{n} ^{\prime}\varphi_{n} ^{\prime} + Ntg_{n} ^{{\prime}{2}} + Qg_{n} ]} \bigg]ds,\,$$

24 $$\varphi_{n + 1} \left( \eta \right)\, = \,\,\varphi_{n} (\eta ) + \int_{0}^{\eta } {\lambda_{\varphi } (s)\bigg[\varphi_{n} ^{\prime\prime} + Le(f\varphi_{n} ^{\prime}) + \frac{Nt}{{Nb}}g_{n} ^{\prime\prime}}\bigg ]ds,\,$$

To find the Lagrange multipliers $\lambda_{g} (s)$ and $\lambda_{\theta } (s),\,$ we first restrict the non-linear terms and then apply the correctional functional $\delta$ on both sides, we obtain the following results:

25 $$\begin{gathered} \lambda_{f} (s) = \frac{{ - (\eta - s)^{2} }}{2!},\quad \lambda_{g} (s) = (\eta - s), \hfill \\ \lambda_{\phi } (s) = (\eta - s), \hfill \\ \end{gathered}$$

We determine the values in given form $Nt = 0.1$,$Nb = 0.5$,$\Pr = 0.71$,$Le = 1.5$,$\beta = 0.1$, $K = 0.5$, $Ec = 0.5$,$R = 0.1$,$L = 0.5$,$Q = 1$.

And using Eq. (11) as a boundary conditions in Eqs. (19) to (22), we obtain

26 $$f_{0} (\eta ) = - \frac{1}{10}\eta + \frac{211}{{2500}}\eta^{2} ,$$

27 $$g_{0} (\eta ) = 1 + \frac{{{1019}}}{1000}\eta$$

28 $$\varphi_{0} (\eta ) = 1 + \frac{{{1053}}}{1000}\eta ,$$

$$f_{1} = 1 - \frac{793}{{10000}}\eta^{3} - \frac{211}{{5000}}\eta^{4} + \frac{29}{{6250}}\eta^{5} - \frac{1}{2500}\eta^{7}$$

$$g_{1} = 1 + \frac{1019}{{1000}}\eta - \frac{1329}{{1000}}\eta^{2} + \frac{{{849}}}{5000}\eta^{3} - \frac{43}{{5000}}\eta^{5} + \frac{17}{{2000}}\eta^{6}$$

$$\varphi_{1} = 1 + \frac{{{1053}}}{1000}\eta - \frac{1053}{{2000}}\eta^{2} + \frac{11}{{1250}}\eta^{5}$$

The final solution of the above equation is as follows:

29 $$\begin{gathered} f(\eta ) = \mathop {\lim }\limits_{n \to \infty } f_{n + 1} \left( \eta \right)\, \hfill \\ g(\eta ) = \mathop {\lim }\limits_{n \to \infty } g_{n + 1} \left( \eta \right)\, \hfill \\ \varphi (\eta ) = \mathop {\lim }\limits_{n \to \infty } \varphi_{n + 1} \left( \eta \right)\, \hfill \\ \end{gathered}$$

## Validity of solution

Table 1 reflects the solution versification by making the comparison with work of Amir et al.^22^, Ibrahim et al.^43^ and Watanabe^44^. A convincing solution accuracy is noted.

**Table 1:** Comparison of results $- f^{\prime\prime}(0)$ and $- \theta ^{\prime}(0)$ for alternate values of *m* for $K = Ec = R = Le = \Pr = M = Nt = \lambda = \beta = \,\,Q = 0\,.$

M	$- f^{\prime\prime}(0)$				$- \theta ^{\prime}(0)$			
	VIM	Amir et al.^22^	Ibrahim et al.^43^	Watanabe^44^	VIM	Amir et al.^22^	Ibrahim et al.^43^	Watanabe^44^
0.0000	0.46982	0.4698	0.4696	0.4696	0.42151	0.4212	0.42016	0.42015
0.0141	0.50495	0.5048	0.50461	0.50461	0.42876	0.4268	0.42578	0.42578
0.0435	0.56944	0.5691	0.56898	0.56898	0.43641	0.4363	0.43548	0.43548
0.0909	0.66433	0.6623	0.65498	0.65498	0.47211	0.4713	0.44730	0.4473
0.1429	0.73670	0.7367	0.732	0.732	0.47923	0.4789	0.45694	0.45693
0.2000	0.80602	0.8052	0.80213	0.80213	0.48645	0.4855	0.46503	0.46503
0.3333	0.92905	0.9291	0.92765	0.92765	0.49733	0.4966	0.47814	0.47814
1.0000	1.23284	1.2328	1.23258		0.52663	0.5196		

## Results and discussion

**Figure 1:**
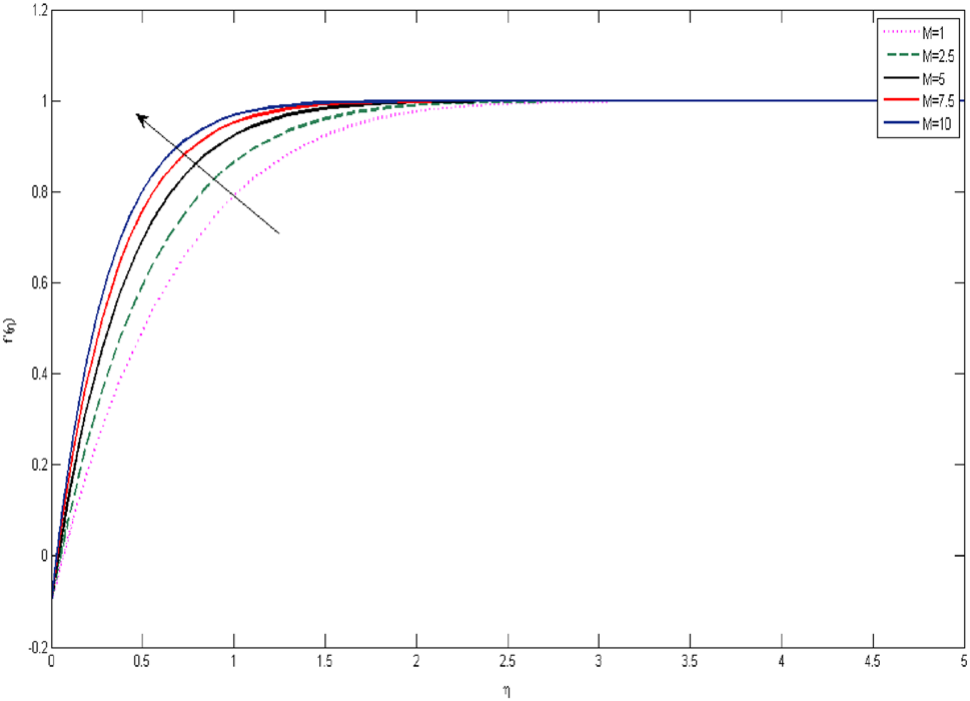
$$M$$ influence on $$f^{\prime}$$.

**Figure 2:**
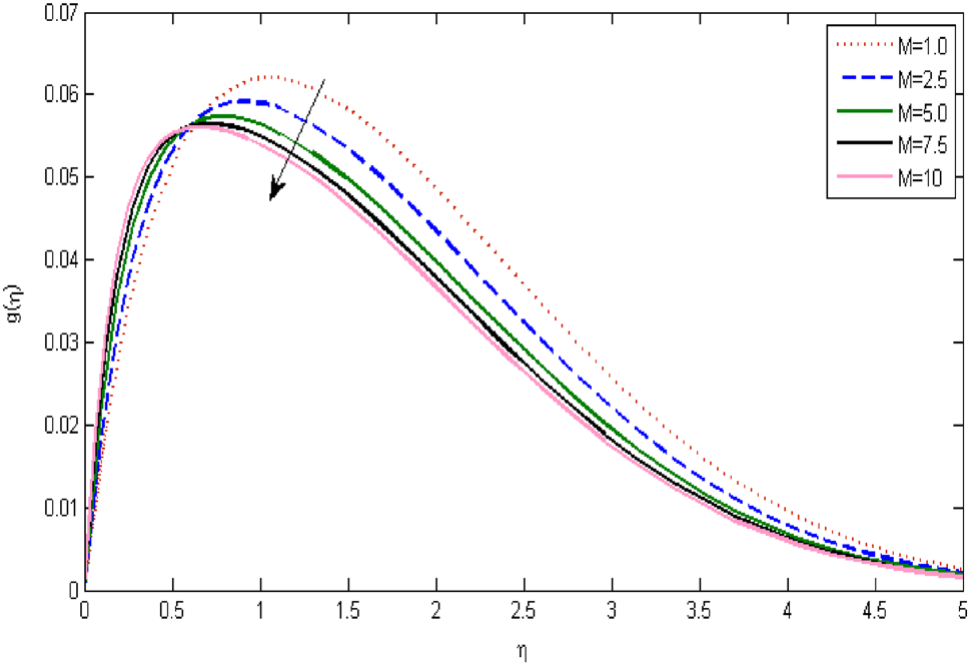
influence on $$g$$.

**Figure 3:**
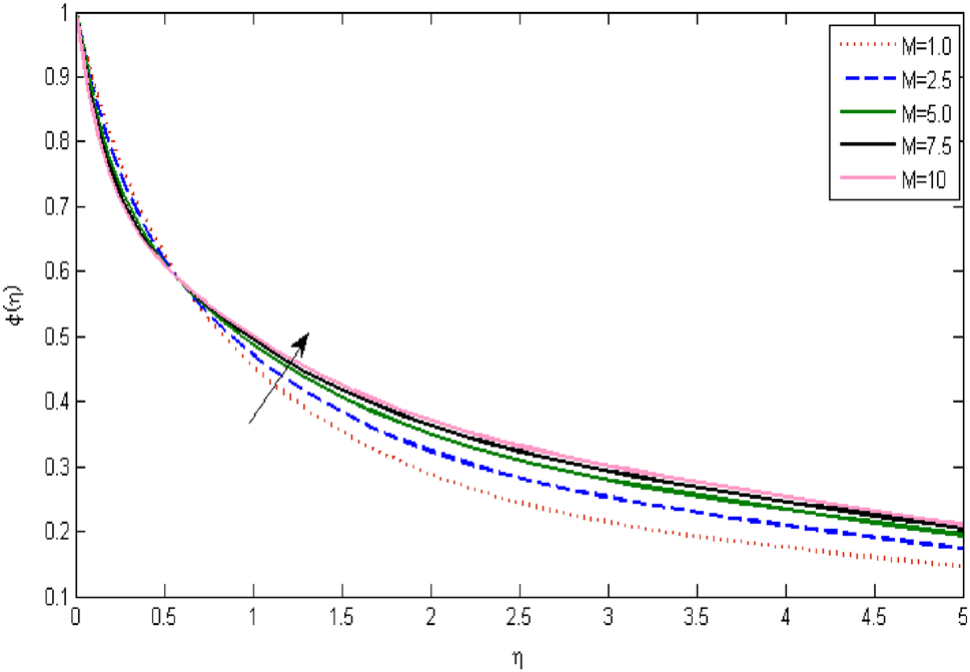
$$M$$ influence on $$\varphi$$.

**Figure 4:**
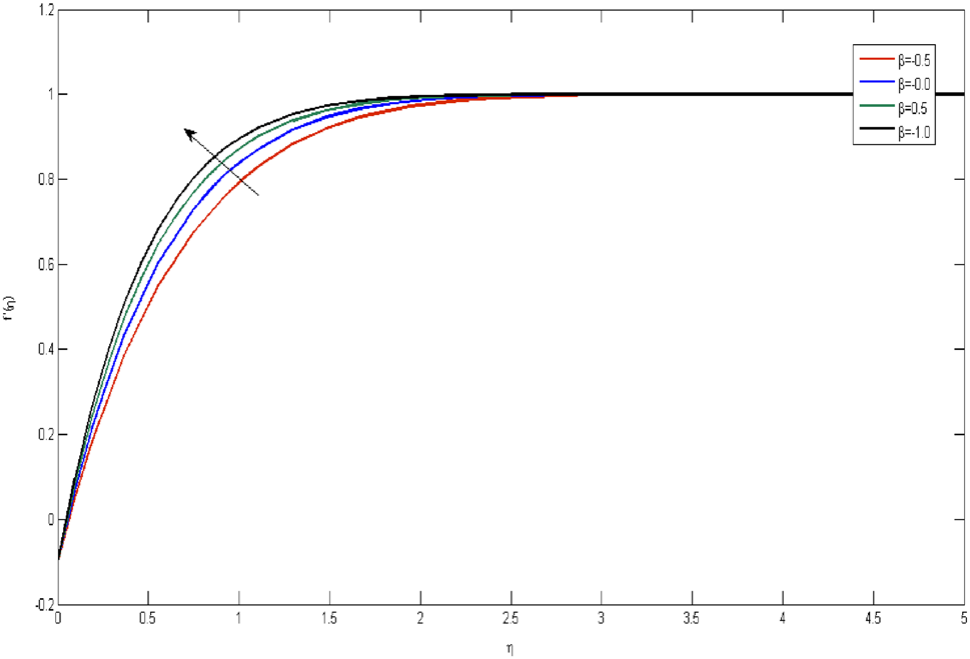
influence on $$f^{\prime}$$.

**Figure 5:**
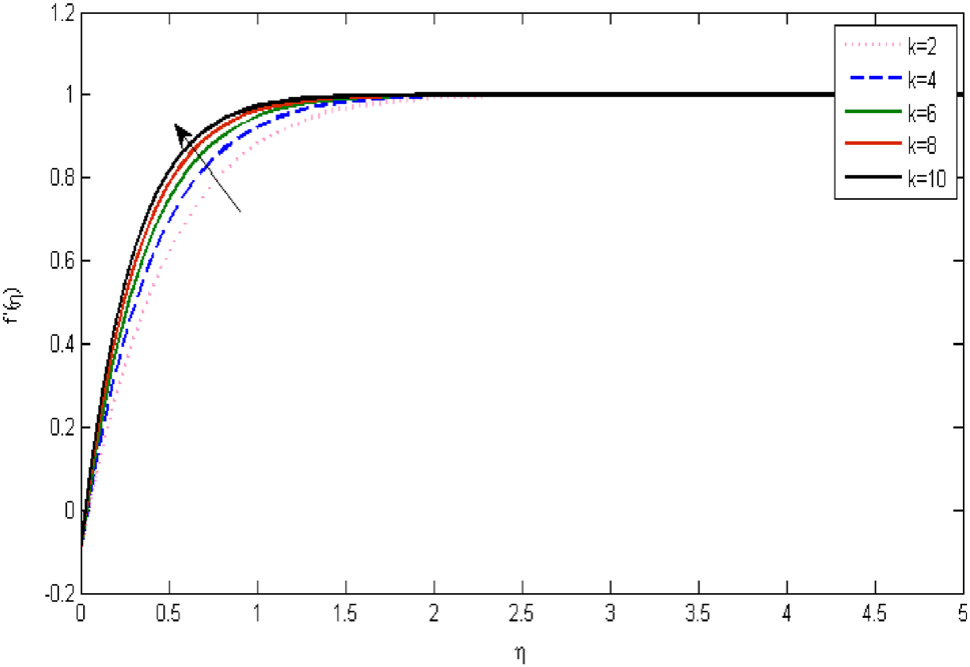
influence on $$f^{\prime}$$.

**Figure 6:**
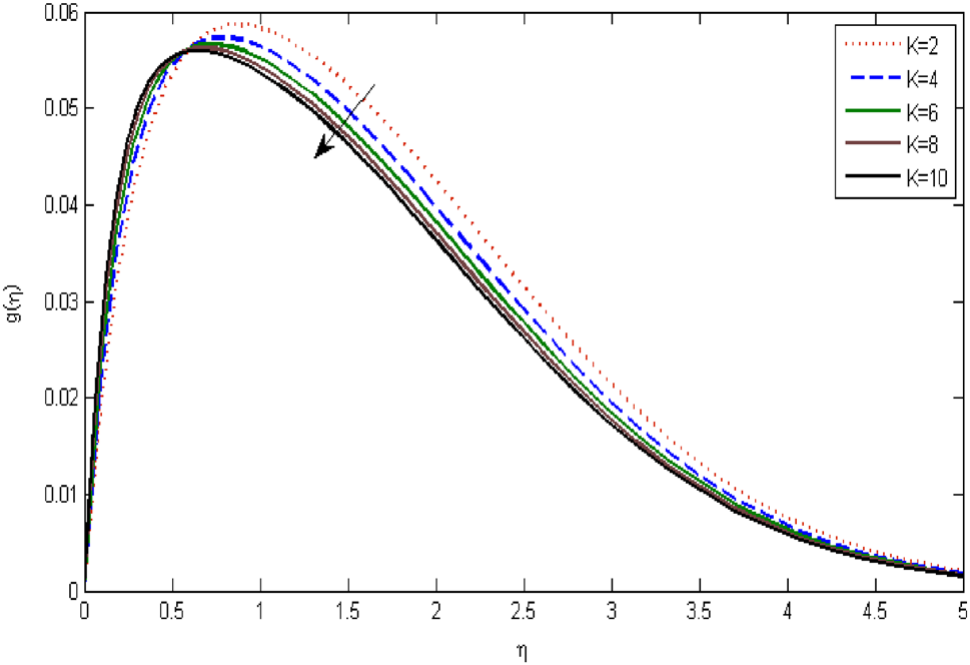
$$K$$ influence on $$g$$.

**Figure 7:**
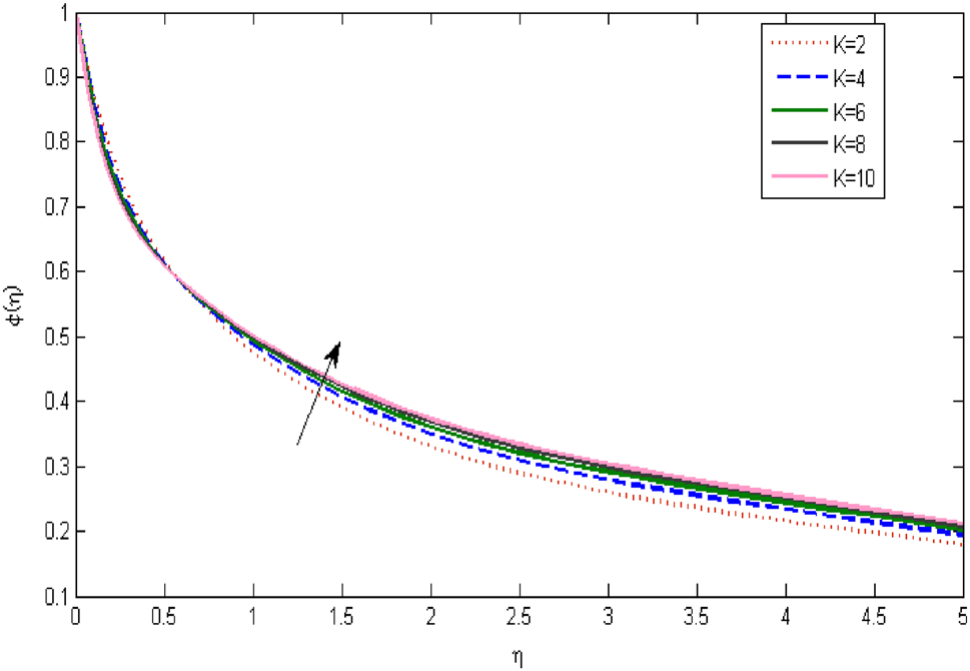
influence on $$g$$.

**Figure 8:**
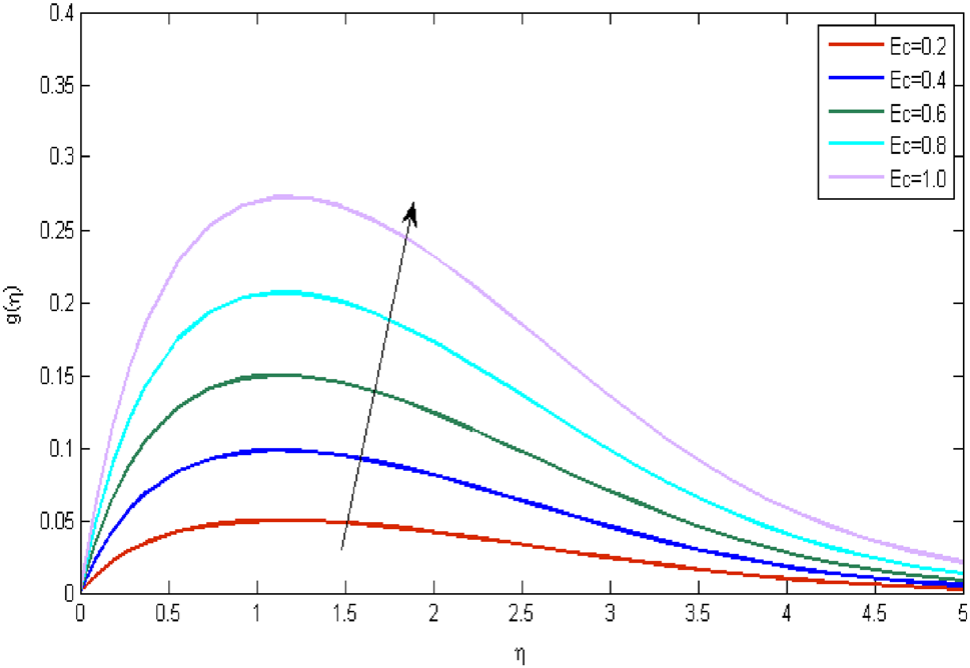
$$Ec$$ influence on $$g$$.

**Figure 9:**
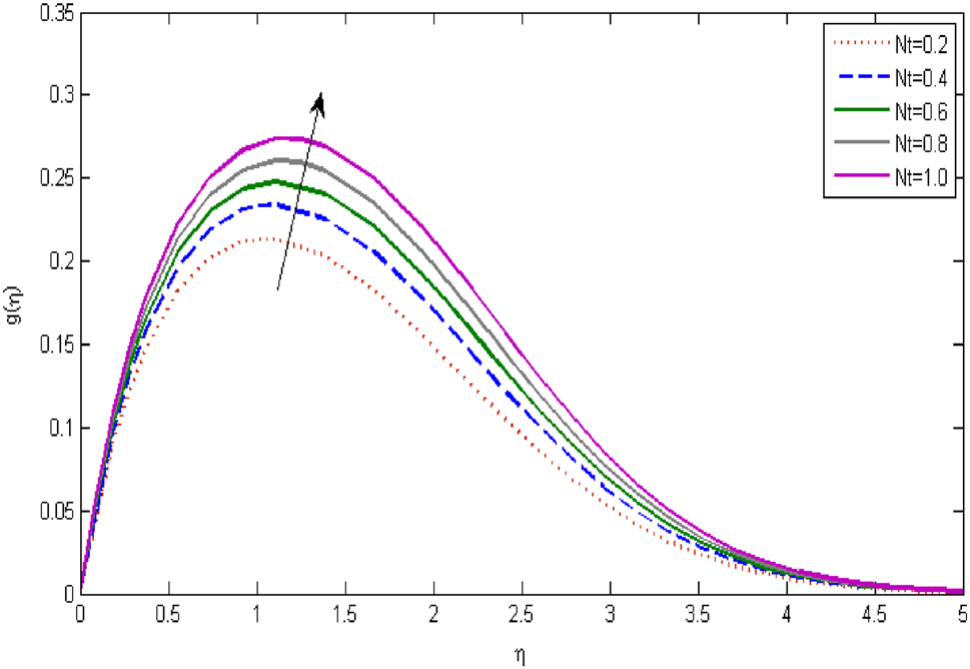
influence on $$g$$.

**Figure 10:**
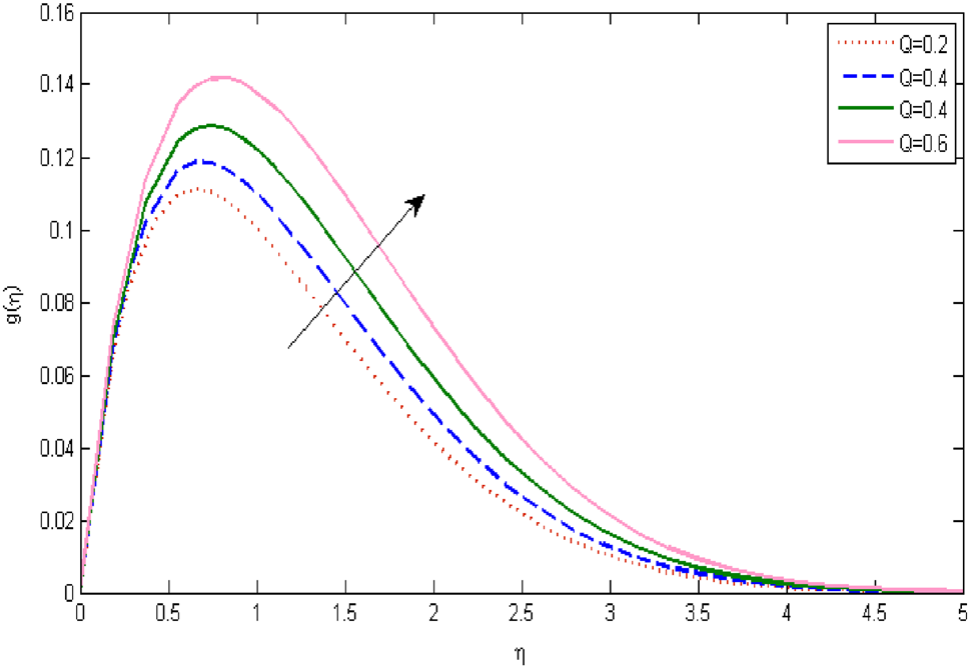
$$Q$$ influence on $$g$$.

**Figure 11:**
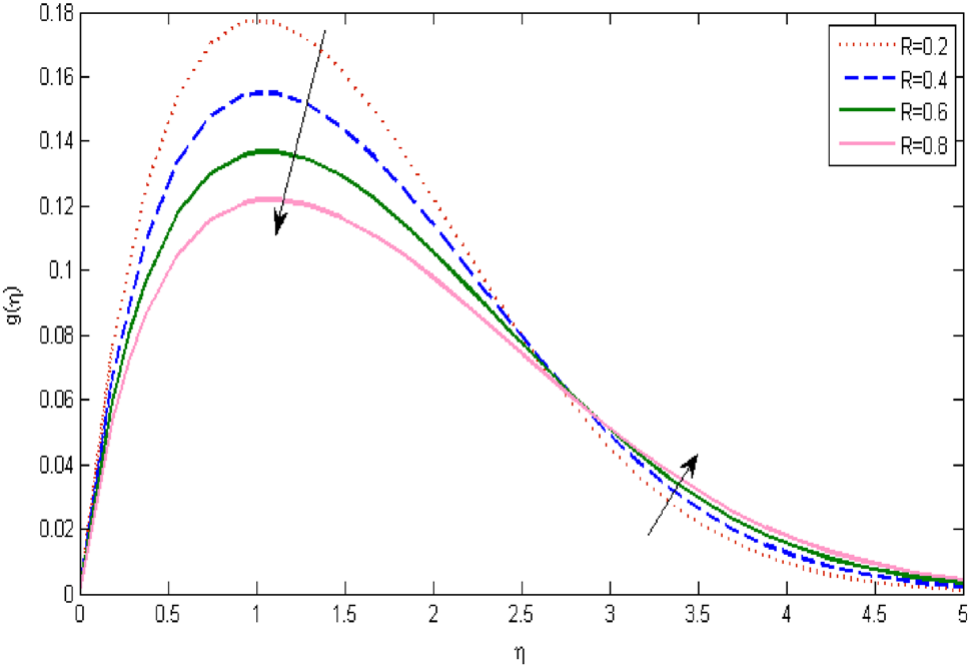
influence on $$g$$.

**Figure 12:**
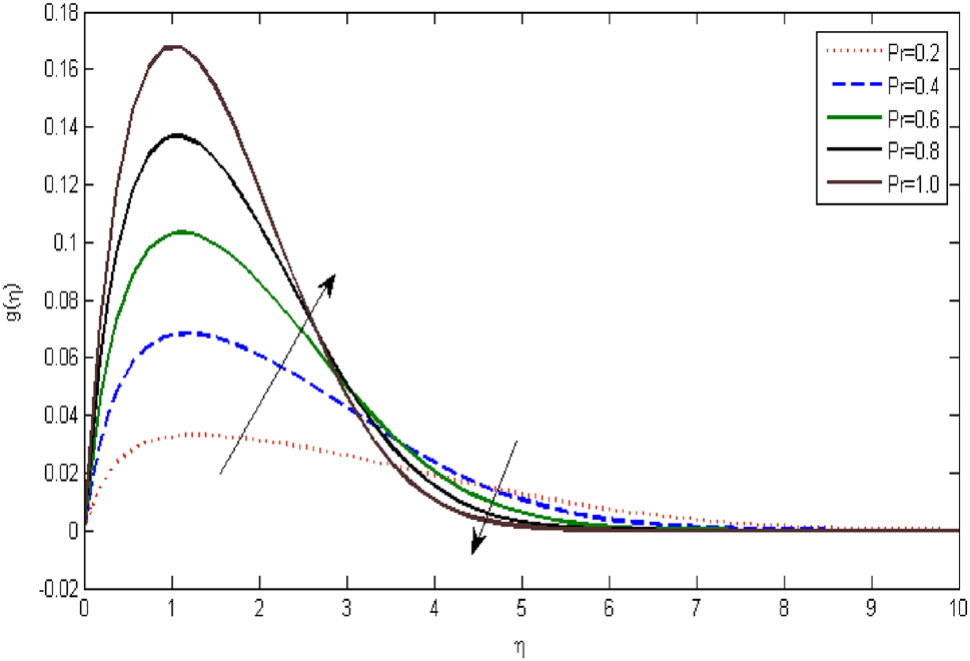
$$\Pr$$ influence on $$g$$.

In the current portion, the physical impact of parameters has been focused. The physical impact of magnetic parameter on velocity profile has been addressed in Fig. 1. The lower trend in velocity is observed with growing values of magnetic parameter. Physically, such observations are due to Lorentz force which operates as a retarding force on the velocity field and its decrease of fluid flow for velocity boundary layer thickness. The Lorentz force *(U* > *u)* is beaten by the pressure force as the magnetic parameter effect climbs the velocity*.* The magnetic parameter effect surges the velocity. In addition, the magnetic parameter impact decreases the flow of velocity, therefore contracts the size of the boundary momentum sheet as well, as the pressure force (*u* > *U*), leaded by Lorentz force. Similarly, it is also portrayed in Figs. 2 and 3 that a growth of magnetic parameter diminishes the thermal and concentration profile. Figure 4 depicts the pressure gradient impacts over curves of velocity. It is apparent that the rise in velocity profile with a growth of the wedge parameter. The flow of fluid is very slow and decreases width of boundary layer owing velocity to increases angle of wedge. Figure 5 depicts the sway of different permeability parameter $K$ for the velocity profile. It is determining that a growth of permeability parameter surges a momentum boundary layer width. Same impact can be seen in Figs. 6 and 7. The results of Eckert number is reported in Fig. 8 for temperature profile. It is clearly observed that a growth in viscous parameter consequence in a rise in the temperature distribution, along decline the concentration profile. Figure 9 are prepared in order to display the habits of the thermophoresis parameter on the temperature. The rise in the temperature is clearly observed for different value of $Nt$. The results disclosed in Fig. 10 expressed the variation in temperature due to $Q$. It can be analyzed that temperature increases as a consequence of a surge in heat source factor. Figure 11 illustrate the temperature profile influences over radiation parameter. Interestingly, it is observed that temperature decreases with the growth of radiation parameter. Figure 12 presents the Prandtl number influence on the temperature rate. A significant decline is shown for temperature profile with growth of Prandtl number. The numerical values of local Nusselt number are signified for different parameters. The enactment of local Nusselt number is noticed for angle of rotation. The skin friction coefficient rises as the growth in magnetic parameter and permeability parameter. The Table 2 displays the numerical aspects of local Sherwood number, coefficient of skin friction and local Nusslet number subject to the following fixed estimations of parameters i.e., *Pr* = 0.71, *c* = 0.5,*Nb* = 0.5,*Nt* = 0.1, *Le* = 0.5, *R* = 0.1, *Q* = 1.

**Table 2:** The skin friction coefficient, Local Nusselt number, and Local Sherwood number alterations for a constant parameters of *Pr* = 0.71, *c* = 0.5, *Nb* = 0.5,*Nt* = 0.1, *Le* = 0.5, *R* = 0.1, *Q* = 1.

$\beta$	$M$	$K$	$2\sqrt {\frac{m + 1}{2}} f^{\prime\prime}(0)$	$2\sqrt {\frac{m + 1}{2}} g^{\prime\prime}(0)$	$2\sqrt {\frac{m + 1}{2}} \theta^{\prime}(0)$
0.1	2	0.5	3.42531	− 0.7105	0.40797
0.5			3.77520	− 0.7995	0.45986
1			4.49837	− 0.9792	0.56407
0.1	1		2.69044	− 0.6823	0.36296
	5		5.02279	− 0.8227	0.47490
	10		6.90843	− 0.9973	0.52523
	2	0.5	3.42531	− 0.7105	0.40797
		1	3.73917	− 0.7283	0.42384
		1.5	4.02870	− 0.7469	0.43710

## Conclusions

The effects of viscous dissipation, thermophoresis Brownian motion, and MHD fluid boundary layer on a wedge embedded in porous medium that uses nanofluid mass and heat transfer were investigated in this study. The PDEs are transformed into a set of nonlinear ODEs using the requisite similarity transformations. The method of variational iteration scheme is implemented. The series solution convergence is demonstrated by graphs. Following are the main conclusion of the present study:

- A declining varaition in velocity is observed due to larger change in magnetic constant.
- The concetration profile enahcned due to magnetic ocnstant.
- The presence of permeabilty of porous space controls the velocity rate.
- With applications of thermal riadation and thermosphresis constant imporved the increasing change in temperautre profile.
- The local Nussel number enahcne due to angle of rotation.

## Data Availability

All the data are clearly available in the manuscript.

## List of symbols

$\lambda$: Wedge stretching rate
$$(u,v)$$: Velocity components
$$g$$: Dimensionless temperature
$$p$$: Pressure
$$\sigma$$: Electrical conductivity
$B_{0}$: Magnetic field strength
$$\rho_{f}$$: Fluid density
$$\beta$$: Wedge angle
$$\psi$$: Stream function
$$C$$: Concentration
$$T$$: Temperature
$$M$$: Magnetic parameter
$$K$$: Porosity parameter
$$R$$: Radiation parameter
EC: Eckert number
$Q$: Heat source constant
$$Nt$$: Thermophoresis parameter
$$Le$$: Lewsis number
$$\Pr$$: Prandtl constant
$$Nb$$: Brownian parameter
${\text{Re}}$: Local Reynold number
$C_{f}$: Skin fraction coefficient
$Nu$: Local Nusselt number
$Sh$: Local Sherwood number
