# Author Correction: Numerical aspects of thermo migrated radiative nanofluid flow towards a moving wedge with combined magnetic force and porous medium

**DOI:** 10.1038/s41598-022-16367-0

**Published:** 2022-07-14

**Authors:** Ehsan Ul Haq, Sami Ullah Khan, Tasawar Abbas, Kamel Smida, Qazi Mahmood Ul Hassan, Bilal Ahmad, M. Ijaz Khan, Kamel Guedri, Poom Kumam, Ahmed M. Galal

**Affiliations:** 1 Department of Mathematics, University of Wah, Wah Cantt, 47040 Pakistan; 2 Department of Mathematics, COMSATS University Islamabad, Sahiwal, 57000 Pakistan; 3 Department of General Sciences, College of Applied Sciences, AlMaarefa University, DiriyahRiyadh, 13713 Saudi Arabia; 4 Department of Mathematics and Statistics, Riphah International University, I-14, Islamabad, 44000 Pakistan; 5 Department of Mechanics and Engineering Science, Peking University, Beijing, China; 6 Mechanical Engineering Department, College of Engineering and Islamic Architecture, Umm Al-Qura University, P.O. Box 5555, Makkah, 21955 Saudi Arabia; 7 Center of Excellence in Theoretical and Computational Science (TaCS-CoE) and KMUTT Fixed Point Research Laboratory, Room SCL 802 Fixed Point Laboratory, Science Laboratory Building, Department of Mathematics, Faculty of Science, King Mongkut’s University of Technology Thonburi (KMUTT), 126 Pracha-Uthit Road, Bang Mod, Thung Khru, Bangkok, 10140 Thailand; 8 Department of Medical Research, China Medical University Hospital, China Medical University, Taichung, 40402 Taiwan; 9 Mechanical Engineering Department, College of Engineering, Prince Sattam Bin Abdulaziz University, Wadi addawaser, 11991 Saudi Arabia; 10 Production Engineering and Mechanical Design Department, Faculty of Engineering, Mansoura University, Mansoura, P.O 35516 Egypt

Correction to: *Scientific Reports* 10.1038/s41598-022-14259-x, published online 16 June 2022

The original version of this Article contained an error in the Acknowledgments section.

"The authors would like to thank the Deanship of Scientific Research at Umm Al-Qura University for supporting this work by Grant Code: 22UQU4331317DSR14. The authors acknowledge the financial support provided by the Center of Excellence in Theoretical and Computational Science (TaCS-CoE), KMUTT. Kamel SMIDA would like to express his gratitude to AlMaarefa University, Riyadh, Saudi Arabia, for providing funding (TUMA-2021-17) to do this research. Moreover, this research project is supported by Thailand Science Research and Innovation (TSRI) Basic Research Fund: Fiscal year 2022 under project number FRB650048/0164."

now reads:

"The authors would like to thank the Deanship of Scientific Research at Umm Al-Qura University for supporting this work by Grant Code: 22UQU4331317DSR16. The authors acknowledge the financial support provided by the Center of Excellence in Theoretical and Computational Science (TaCS-CoE), KMUTT. Kamel SMIDA would like to express his gratitude to AlMaarefa University, Riyadh, Saudi Arabia, for providing funding (TUMA-2021-17) to do this research. Moreover, this research project is supported by Thailand Science Research and Innovation (TSRI) Basic Research Fund: Fiscal year 2022 under project number FRB650048/0164."

The original Article has been corrected.

